# Detection of Low Pathogenicity Influenza A(H7N3) Virus during Duck Mortality Event, Cambodia, 2017

**DOI:** 10.3201/eid2406.172099

**Published:** 2018-06

**Authors:** Annika Suttie, Sokhoun Yann, Phalla Y, Sothyra Tum, Yi-Mo Deng, Vibol Hul, Viseth Srey Horm, Ian Barr, Andrew Greenhill, Paul F. Horwood, Kristina Osbjer, Erik A. Karlsson, Philippe Dussart

**Affiliations:** Institut Pasteur du Cambodge, Phnom Penh, Cambodia (A. Suttie, S. Yann, P. Y, V. Hul, V.S. Horm, E.A. Karlsson, P. Dussart);; Federation University, Churchill, Victoria, Australia (A. Suttie, A. Greenhill);; National Animal Health and Production Research Institute, Cambodia Ministry of Agriculture, Forestry and Fisheries, Phnom Penh (S. Tum);; World Health Organization Collaborating Centre for Reference and Research on Influenza, Melbourne, Victoria, Australia (Y.-M. Deng, I. Barr);; James Cook University, Cairns, Queensland, Australia (P.F. Horwood); Food and Agriculture Organization of the United Nations, Phnom Penh (K. Osbjer)

**Keywords:** low pathogenicity, influenza, H7N3, LPAI, LPAIV, AIV, avian, poultry, ducks, mortality event, Cambodia, zoonoses, viruses

## Abstract

In January 2017, an estimated 3,700 (93%) of 4,000 Khaki Campbell ducks (*Anas platyrhynchos domesticus*) died in Kampong Thom Province, Cambodia. We detected low pathogenicity avian influenza A(H7N3) virus and anatid herpesvirus 1 (duck plague) in the affected flock; however, the exact cause of the mortality event remains unclear.

Avian influenza viruses (AIVs) are negative-sense, single-stranded RNA viruses normally found in wild aquatic birds, the natural reservoir (*1*). Typically, AIVs do not cause severe disease in domestic poultry; however, 2 AIV subtypes, H5 and H7 influenza A viruses, are capable of mutating to form highly pathogenic avian influenza (HPAI) variants that can cause high rates of disease and death in poultry flocks (*2*). In addition, establishment of AIVs in domestic poultry increases the probability of zoonotic transmission to humans. Recent attention has focused on H7 AIVs (particularly subtype H7N9 in China) that have become established in domestic poultry and repeatedly transmitted to humans since 2013 (*3*). Influenza A(H7N3) and A(H7N7) viruses have also been the causative agent in historical poultry and human infections in Europe and the Americas (*2*). Overall, the pathogenic and zoonotic potential of H7 strains makes them a substantial economic and public health concern.

Cambodia is a lower-middle-income country in Southeast Asia with a large socioeconomic dependence on agriculture. In 2015, a total of 57% of all households in Cambodia had agricultural holdings, and 87% of these households raised poultry (*4*). Poultry are generally reared in backyards or on small-scale farms with minimal or no biosecurity. Therefore, poultry diseases such as HPAI can have devastating economic consequences. In 2013 alone, ≈25 million chickens and 3.3 million ducks were either traded or disposed of (slaughtered for sale or died) in Cambodia. Of these, 22% of chickens and 18% of ducks were reported to have died from illness (*5*). Although control measures for HPAI in Cambodia include culling of poultry that is infected, suspected to be infected, or in contact with infected/suspected poultry, reporting is minimal, and no compensation mechanism exists. Since 2004, a total of 58 reported AIV outbreaks (mostly HPAI) have occurred in poultry and wild birds (as of April 2018), and 56 human influenza A(H5N1) cases (37 fatalities [case-fatality rate 66%]) have been reported in Cambodia (*6*).

## The Study

**Figure 1:**
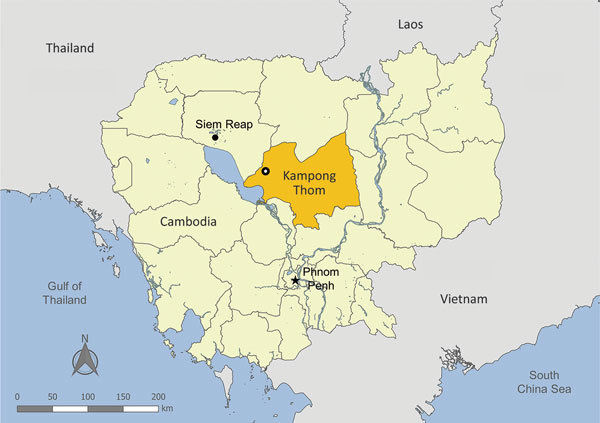
Location of duck mortality event and detection of influenza A(H7N3) virus in Kampong Thom Province (gold shading), Cambodia, January 2017. Open circle indicates exact location of the mortality event.

In early January 2017, a free-range production flock of 4-month-old Khaki Campbell ducks located in Kampong Thom Province in central Cambodia were found with loss of appetite, depression, weakness, white-bluish diarrhea, and swollen heads and eyes (Figure 1). Within days, ≈93% (an estimated 3,700 of 4,000) had succumbed to disease. The remainder of the flock was slaughtered. As part of a routine investigation into the causative agent, we obtained oropharyngeal and cloacal swab specimens and organs from 4 of the affected ducks. The National Animal Health and Production Research Institute of Cambodia performed initial screening for the presence of AIV by real-time quantitative reverse transcription PCR (qRT-PCR). The Institut Pasteur du Cambodge (IPC) verified the results and conducted further analysis.

IPC confirmed by qRT-PCR that 2 of 4 ducks were positive for influenza A virus. IPC successfully isolated viruses from both samples in embryonated chicken eggs (*7*) and designated them A/duck/Cambodia/b0116502/2017 and A/duck/Cambodia/b0120501/2017. Next, the World Health Organization’s Collaborating Centre for Reference and Research on Influenza (Melbourne, VIC, Australia) performed whole-genome sequencing on isolate RNA using the Ion Torrent next-generation sequencing (NGS) platform (Life Technologies, Carlsbad, CA, USA) and analyzed NGS data by using CLC Genomic Workbench 10 (https://www.qiagenbioinformatics.com/products/clc-genomics-workbench). Sanger sequencing with segment-specific primers filled in any sequencing gaps (Technical Appendix Table 1) using Big Dye Terminator Reaction Mix (Applied Biosystems, Foster City, CA, USA) on an ABI 3500xL Genetic Analyzer (Applied Biosystems). IPC, in conjunction with the World Health Organization’s Collaborating Centre for Reference and Research on Influenza, used Geneious 9.1.8 (Biomatters Ltd., Auckland, New Zealand) to collate NGS and Sanger sequencing data, align strains, and analyze molecular markers. IPC submitted all sequences to GenBank (accession nos. MG591682–MG591697; Technical Appendix Table 2). We used the maximum-likelihood method based on the general time-reversible model to infer phylogenic relationships and tree construction for each gene in MEGA version 7 (*8*) with 500 bootstrap replicates for robustness.

**Figure 2:**
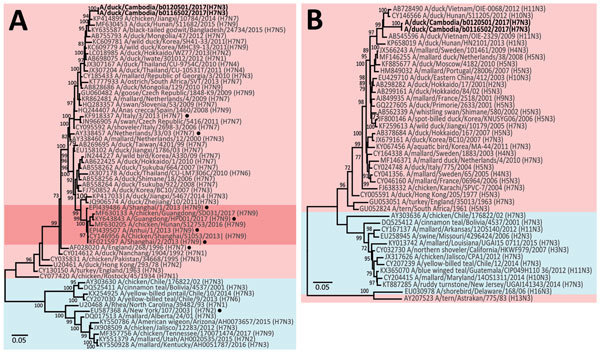
Phylogenetic analysis of the H7 hemagglutinin (HA) gene (A) and N3 neuraminidase (NA) gene (B) of influenza viruses isolated from ducks in Kampong Thom Province, Cambodia (boldface), and reference isolates. Trees were generated using the maximum-likelihood method based on the general time-reversible model. Bootstrap values (n = 500) >70 are indicated. Light pink shading indicates strains from Eurasia, blue shading indicates strains from North and South America, and dark pink shading indicates H7N9 strains from China. Black dots indicate strains from human infections. GenBank accession numbers are provided for reference isolates. Scale bars indicate nucleotide substitutions per site.

Sequencing revealed that both isolates belonged to the H7N3 subtype. Identification of H7 is not novel in Cambodia. Prior studies in 2013 and 2015 in live bird markets have identified low pathogenicity AIV (LPAIV) subtype H7 circulating in chickens and ducks (*6*,*7*). Phylogenetic analyses indicated that all of the gene segments from both H7N3 isolates from Cambodia showed the highest degree sequence similarity to each other and fell into the Eurasian lineage of H7 viruses circulating in Asia, predominantly during 2012–2015. Neither strain shared genes with H7N9 viruses associated with human cases in China (Table 1; Figure 2; Technical Appendix Figure 1–6). On a molecular level, we determined both isolates to be LPAIVs, showing a monobasic cleavage site, avian receptor specificity, and genetic indications of susceptibility to neuraminidase and matrix protein ion channel inhibitors (Table 2).

**Table 1:** Sequence similarity between influenza A virus subtype H7N3 isolates and other influenza viruses, Cambodia*

*Gene segment*	*Strain with highest sequence identity*	*Position, nt*	*Identity, %*	*GenBank accession no.*
*PB2*	A/duck/Hunan/S11682/2015(H7N9)	1–2280	97	MF630450
*PB1*	A/duck/Hunan/S11682/2015(H7N9)	1–2274	97	MF630451
*PA*	A/duck/Jiangxi/15867/2013(H10N3)	1–2151	97	KP285492
*HA*	A/wild bird/Jiangxi/34458/2013(H7N7)	1–1683	97	KP417103
*NP*	A/duck/Nha Trang/84/2014(H6N6)	1–1497	98	LC050632
*NA*	A/duck/Vietnam/OIE-2329/2009(H11N3)	4–1413	94	AB545596
*MP*	A/duck/Hunan/S4443/2011(H11N9)	1–982	99	CY146719
*NS*	A/duck/Vietnam/HU1–16/2014(H11N7)	1–838	98/99	LC070024

*HA, hemagglutinin; MP, matrix protein; NA, neuraminidase; NP, nucleoprotein; NS nonstructural; PA, polymerase; PB1, polymerase basic protein 1; PB2, polymerase basic protein 2.

**Table 2:** Genetic risk characteristics of influenza A virus subtype H7N3 isolates, Cambodia*

Gene segment and risk factor	Amino acid change	Isolates in Cambodia	Conclusions†	Reference
PB2				
Mammalian host range marker and increased viral pathogenicity	E627K	E	Avian specificity	(*9*)
	D701N	D		
HA				
Multibasic cleavage site causing increased pathogenicity	Multibasic	PEPPKGR/GLF	Monobasic	(*10*)
Increased mammalian receptor specificity	Q226L‡	Q	Avian specificity	(*11*)
	G228S‡	G		
NA				
Resistance to NA inhibitor antivirals	H275Y§	H	Sensitive to oseltamivir	(*12*)
	E119K¶	E		
	R292K	R		
MP				
Resistance to M2 inhibitor antivirals	L26F	Q	Sensitive to M2 inhibitors	(*13*)
	V27A	R		
	A30T	D		
	S31N	V		
	G34E	G		

*HA, hemagglutinin; MP, matrix protein; MP2, matrix protein 2; NA, neuraminidase; PB2, polymerase basic protein 2. †Receptor binding specificity and antiviral sensitivity is predicted based on sequence information and has not been experimentally confirmed. ‡H3 numbering. §N1 numbering. ¶N2 numbering.

Because molecular data indicated that the H7N3 strains in this event were LPAIVs, these viruses were probably not the sole causative agent for such high mortality during this outbreak. However, infection with LPAIV H7N3 might have contributed to lethality by increasing susceptibility to secondary infections. In an effort to identify other possible pathogens, we screened swab samples and internal organs of the same ducks from the outbreak for the presence of anatid herpesvirus 1 (AnHV-1), commonly known as duck plague (*9*). Two of the 3 carcasses screened were positive for AnHV-1 in the liver; however, direct comparisons to swab samples cannot be made because no information was available on the correlation between swab samples and duck carcasses. AnHV-1 is known to cause high mortality in duck flocks globally, including in Cambodia, and co-infection with H7N3 virus and AnHV-1 might have contributed to the outbreak (*10*). From swab samples, we confirmed co-infection with AnHV-1 in the 2 H7N3 virus–positive ducks and 1 of the 2 H7N3 virus–negative ducks; however, our outbreak investigation revealed that ducks were retrospectively vaccinated against AnHV-1 with live attenuated vaccine once the flock began to show signs of illness. Consequently, because AnHV-1 vaccine can be detected by qRT-PCR up to 6 days postvaccination (*9*) and no specific date was available for vaccination before sample collection, no direct conclusions can be made about the associated contribution of AnHV-1 and H7N3 to the mass mortality during this outbreak. The extent and effect of such a co-infection need to be investigated further.

## Conclusions

Given the endemicity of AIVs in Southeast Asia, especially in Cambodia, understanding the prevalence and effect of AIV in the region is vital. Although the H7 viruses identified during this outbreak were determined to be low pathogenicity and their role as causative agents of duck mortality remains unclear, continued active and passive surveillance, as well as molecular characterization and risk assessment, is crucial to identify, control, and prevent AIVs in this region. Further work is also necessary to understand the interplay of AIVs with other diseases of poultry to determine the etiology of bird mortality events in the region.

Technical Appendix Specific primers used to amplify influenza A virus subtype H7N3 genes, GenBank accession numbers, and phylogenetic analysis of internal genes of H7N3 isolates, Cambodia.
