# Supplementary material for: Detection of Low Pathogenicity Influenza A(H7N3) Virus during Duck Mortality Event, Cambodia, 2017
Source: Emerg Infect Dis. 2018 Jun;24(6):1103–7. doi: 10.3201/eid2406.172099 (PMC6004859; doi:10.3201/eid2406.172099)
Supplement: Technical Appendix — Specific primers used to amplify influenza A virus subtype H7N3 genes, GenBank accession numbers, and phylogenetic analysis of internal genes of H7N3 isolates, Cambodia. [file 17-2099-Techapp-s1.pdf]

Article DOI: https://doi.org/10.3201/eid2406.172099

Detection of Low Pathogenicity Influenza Virus A(H7N3) during Duck Mortality Event, Cambodia, 2017

Technical Appendix

Technical Appendix Table 1. Segment specific primers used to amplify H7N3 genes

Gene Segment and Primer ID	Sequence of Primer Pairs 5′ to 3′
PB2	
Pb2f5nc	GCA GGT CAA ATA TAT TCA ATA TGG AGA G
Pb2r960	GCA TAT ATC CAC AGC TTG TTC
Pb2f770	CAG AGT YTG ATC ATH GCT GC
Pb2a1621	TCC CAC ATC ATT GAK GAT GA
PB2F1143	GAT TCA RTT GAT AGT AAG TGG AA
PB2R2341	ATA TGG TCT CGT ATT AGT AGA AAC AAG GTC GTT T
PB1	
Szpb1+	AGC AAA AGC AGG CAA ACC AT
Pb1r1324	TCC GTC CCA CCA ATA TGT GG
Pb1f1230	ACT GAG TCC TGG CAT GAT GAT GGG
Pb1/-/5	TTC ACT ATT TTT GCC GTC TGA GTT CTT C
Pb1f913	TGA CTA ACT CAC CAA GAT ACT G
Pb1r1654	CTG TTG CTG GTC CAA GGT C
PA	
PAUF	AGC AAA AGC AGG TAC TGA T
PA-1168	CTA ACA TCT TTG CAG TCC TC
PAF587	TGG GAT TCC TTT CGT CAA TC
PA1498R	TNG TYC TRC AYT TGC TTA TCA T
PAF1151	CTC GGT GAG AAC ATG GCA C
PAR2234	TGG ACA GTA TGG ATA GCA A
NS	
NSF1	TAT TCG TCT CAG GGA GCA AAA GCA GGG TG
NS890R	ATA TCG TCT CGT ATT AGT AGA AAC AAG GGT GTT TT
NP	
NPF859	AGT GGA TAT GAC TTT GAG AGA GAA
NPR1565	ATA TCG TCT CGT ATT AGT AGA AAC AAG GGT ATT TTT

Technical Appendix Table 2. GenBank accession numbers of Cambodian H7N3 isolates

Virus	Segment Number	Gene Segment	Accession Number
A/duck/Cambodia/b0120501/2017	1	PB2	MG591696
	2	PB1	MG591695
	3	PA	MG591693
	4	HA	MG591683
	5	NP	MG591689
	6	NA	MG591687
	7	M	MG591685
	8	NS	MG591691
A/duck/Cambodia/b0116502/2017	1	PB2	MG591697
	2	PB1	MG591694
	3	PA	MG591692
	4	HA	MG591682
	5	NP	MG591688
	6	NA	MG591686
	7	M	MG591684
	8	NS	MG591690

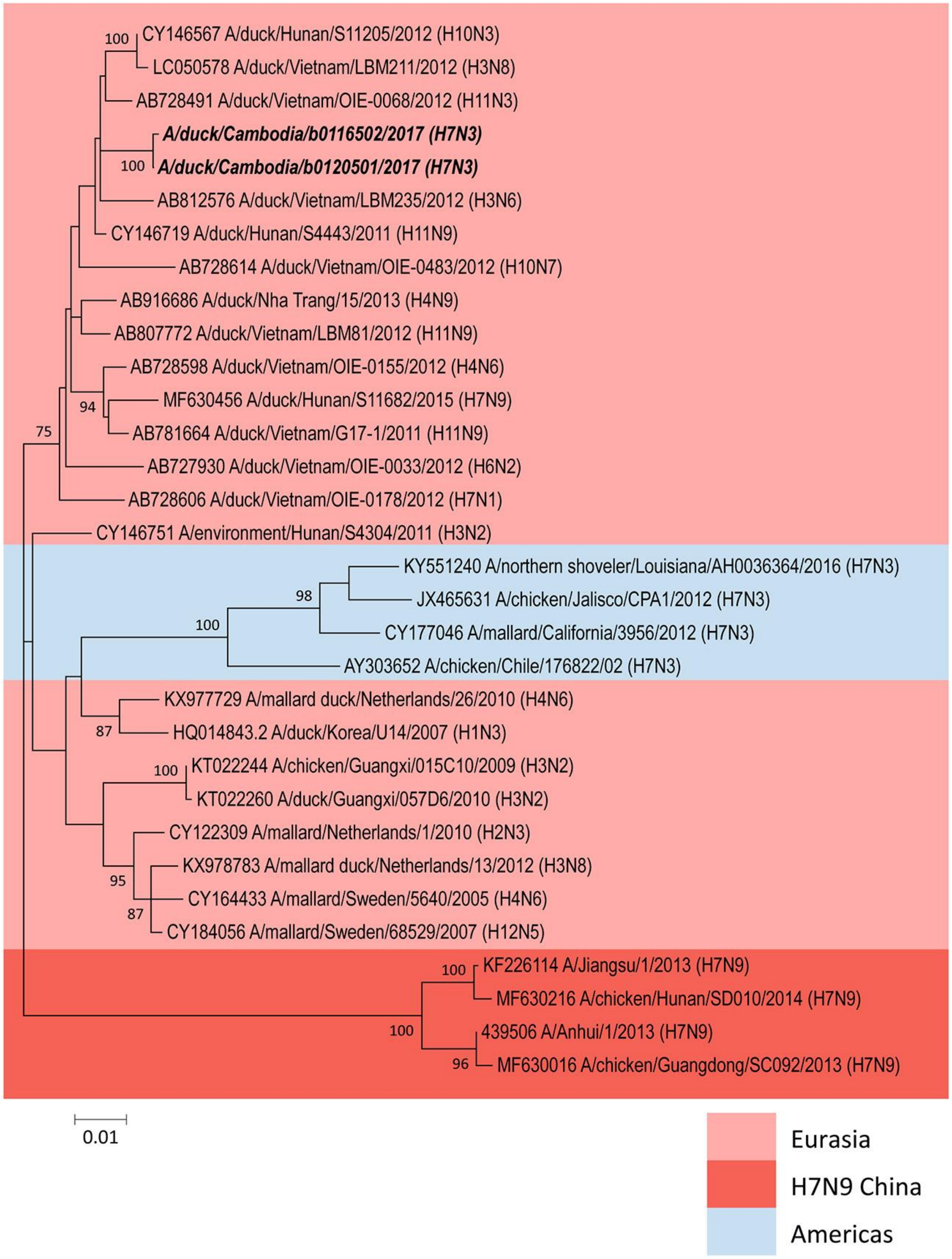

Technical Appendix Figure 1. Phylogenetic analysis (982 nt) of the matrix (MP) gene of viruses isolated from ducks in Kampong Thom province, Cambodia. Trees were generated using the Maximum Likelihood method based on the General Time Reversible model. Scale bars indicate substitutions per site. Bootstrap values (n = 500) >70 are indicated. Cambodian isolates indicated in bold italic. North and South American strains, blue; Eurasian strains, light pink; H7N9 strains from China, red.

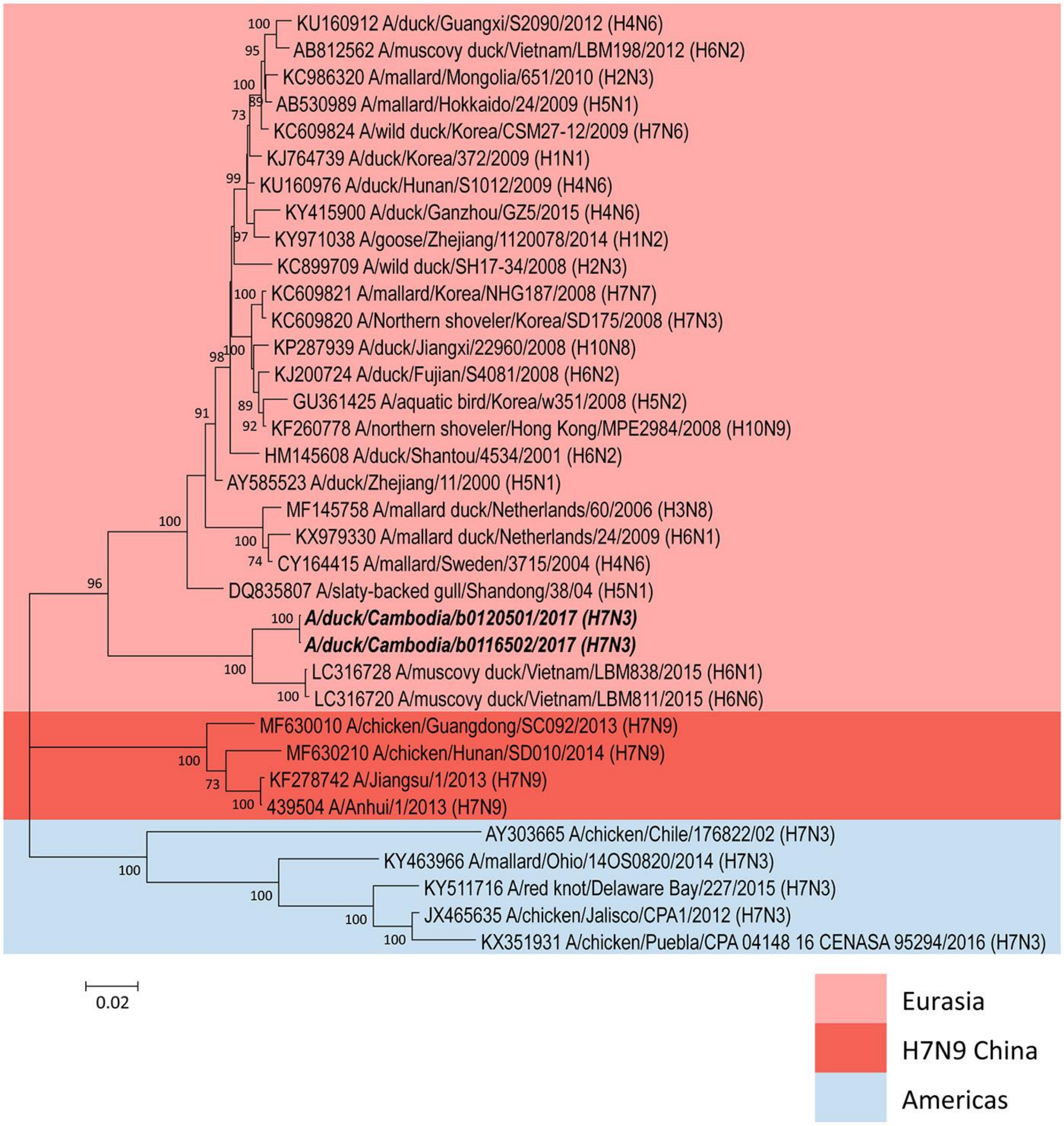

Technical Appendix Figure 2. Phylogenetic analysis (2,280 nt) of the polymerase basic protein 2 (PB2) gene of viruses isolated from ducks in Kampong Thom province, Cambodia. Trees were generated using the Maximum Likelihood method based on the General Time Reversible model. Scale bars indicate substitutions per site. Bootstrap values (n = 500) >70 are indicated. Cambodian isolates indicated in bold italic. North and South American strains, blue; Eurasian strains, light pink; H7N9 strains from China, red.

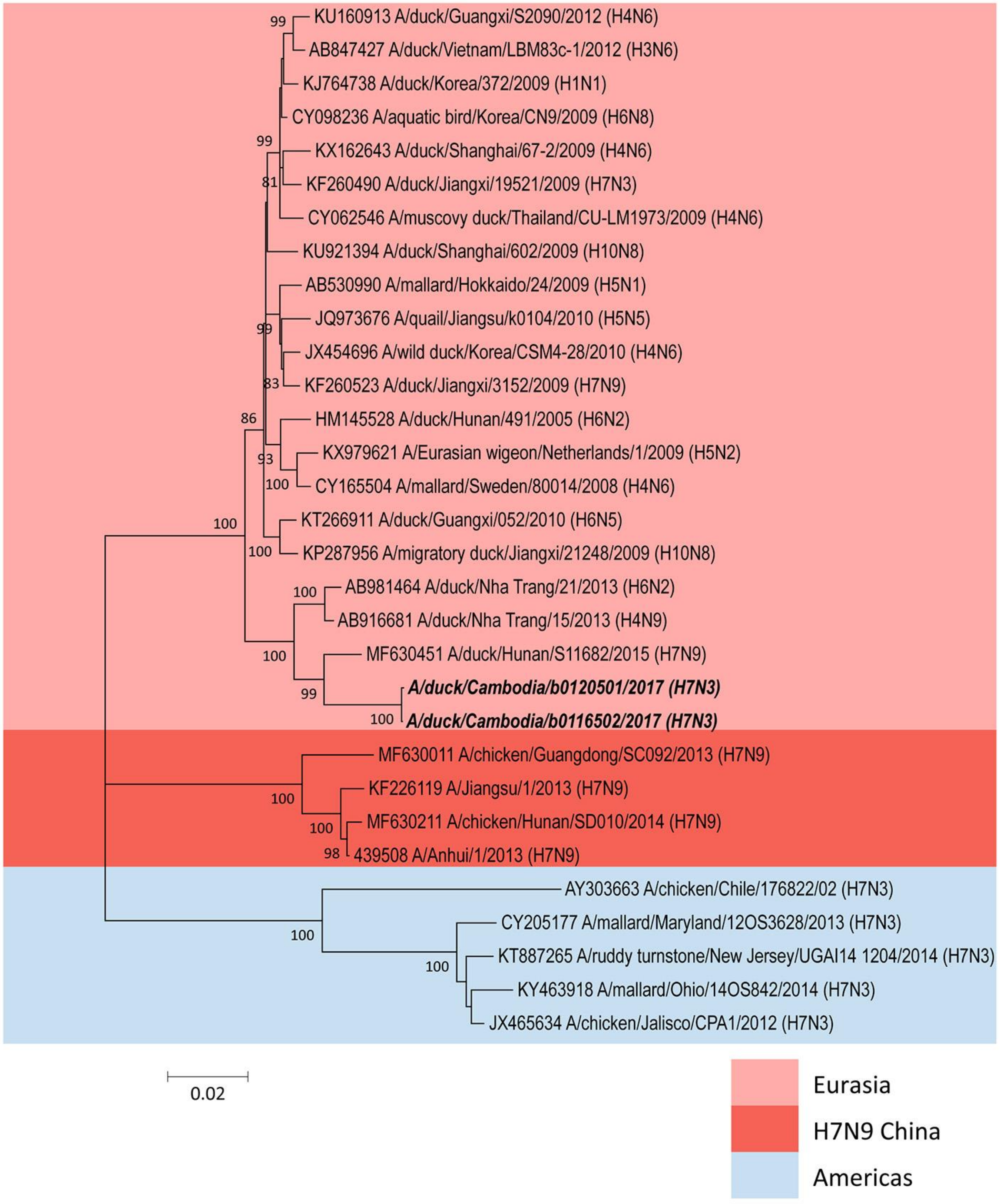

Technical Appendix Figure 3. Phylogenetic analysis (2,274 nt) of the polymerase basic protein 1 (PB1) gene of viruses isolated from ducks in Kampong Thom province, Cambodia. Trees were generated using the Maximum Likelihood method based on the General Time Reversible model. Scale bars indicate substitutions per site. Bootstrap values (n = 500) >70 are indicated. Cambodian isolates indicated in bold italic. North and South American strains, blue; Eurasian strains, light pink; H7N9 strains from China, red.

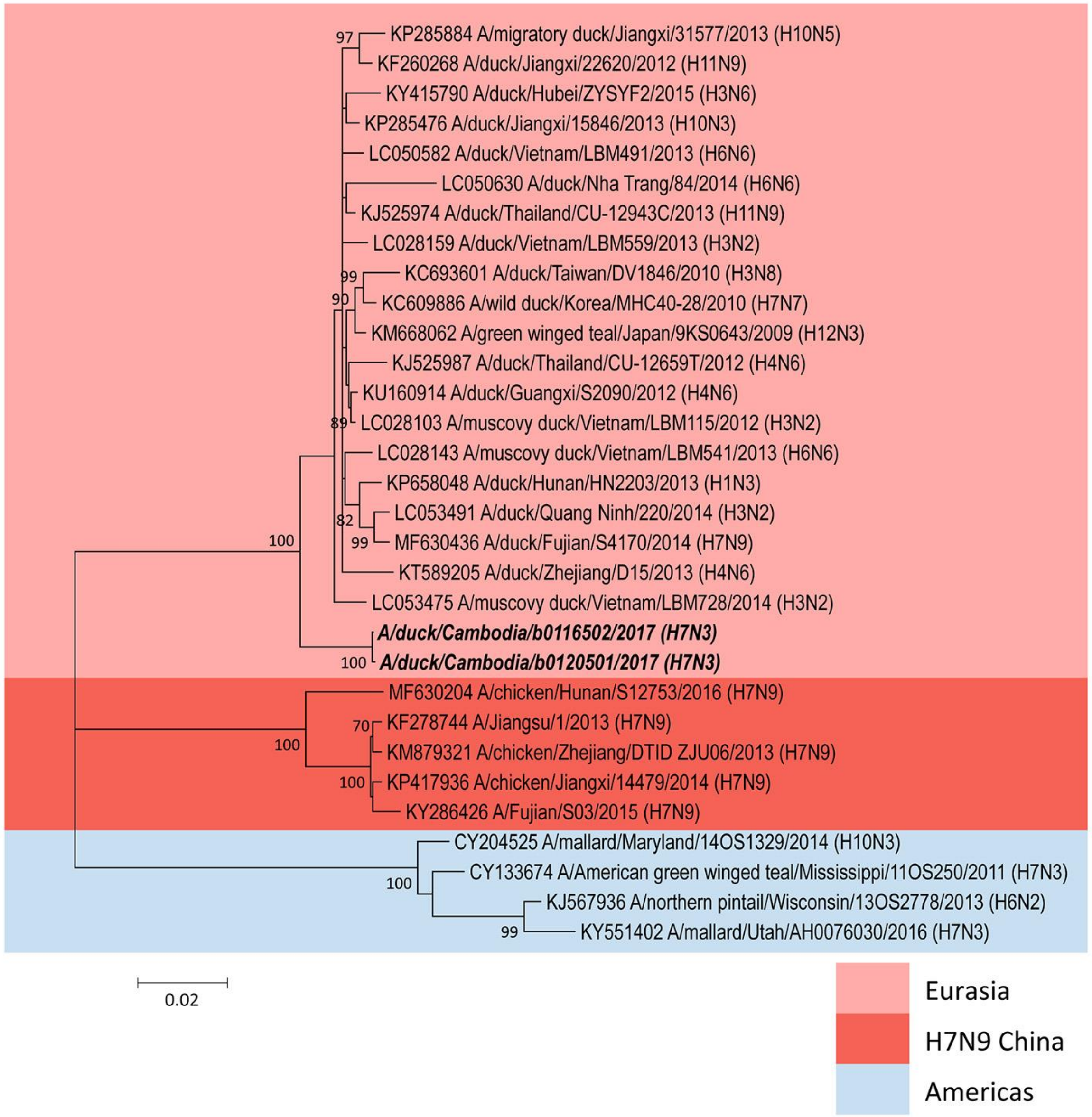

Technical Appendix Figure 4. Phylogenetic analysis (2,151 nt) of the polymerase (PA) gene of viruses isolated from ducks in Kampong Thom province, Cambodia. Trees were generated using the Maximum Likelihood method based on the General Time Reversible model. Scale bars indicate substitutions per site. Bootstrap values (n = 500) >70 are indicated. Cambodian isolates indicated in bold italic. North and South American strains, blue; Eurasian strains, light pink; H7N9 strains from China, red.

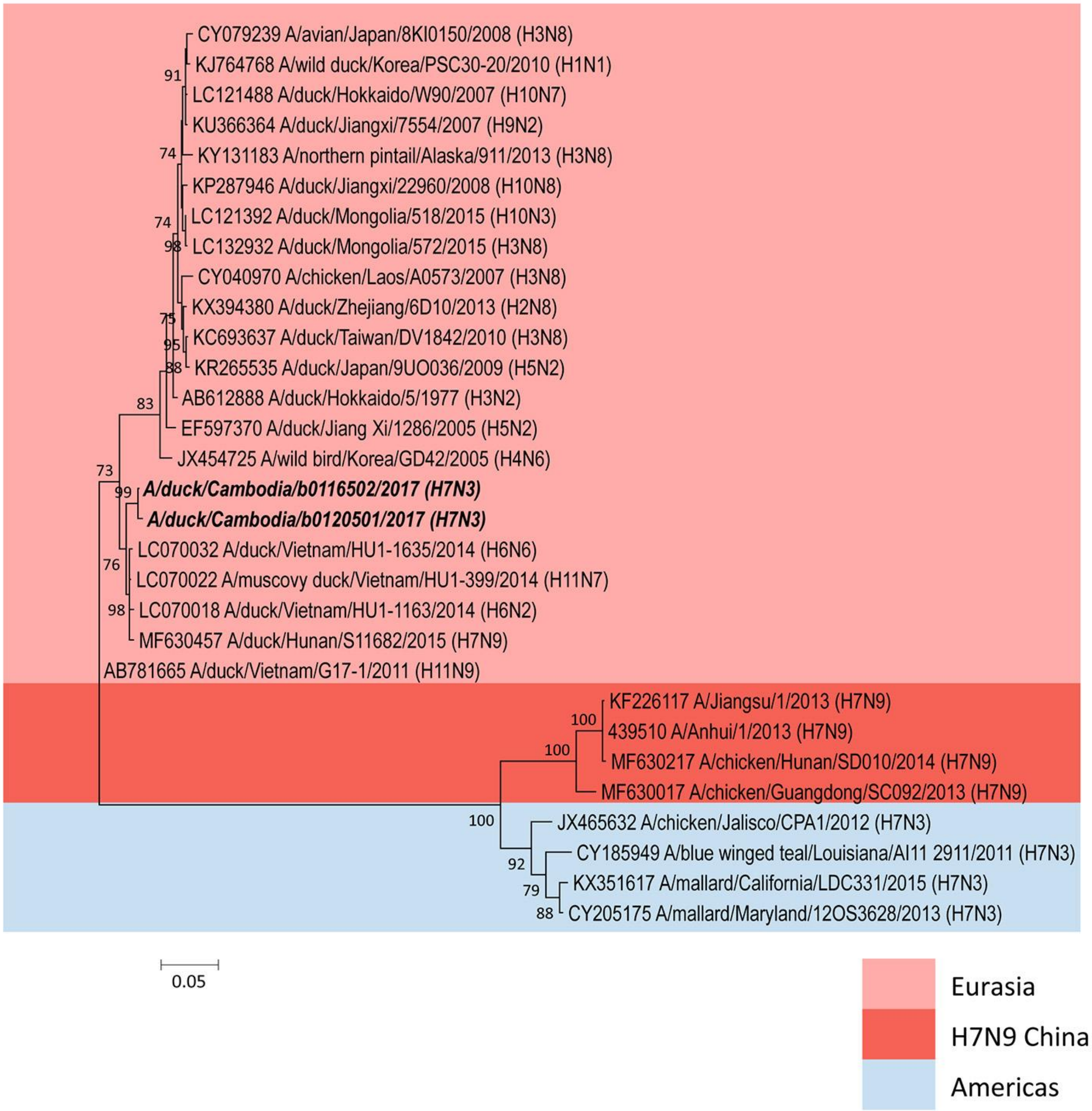

Technical Appendix Figure 5. Phylogenetic analysis (838 nt) of the nonstructural (NS) gene of viruses isolated from ducks in Kampong Thom province, Cambodia. Trees were generated using the Maximum Likelihood method based on the General Time Reversible model. Scale bars indicate substitutions per site. Bootstrap values (n = 500) >70 are indicated. Cambodian isolates indicated in bold italic. North and South American strains, blue; Eurasian strains, light pink; H7N9 strains from China, red.

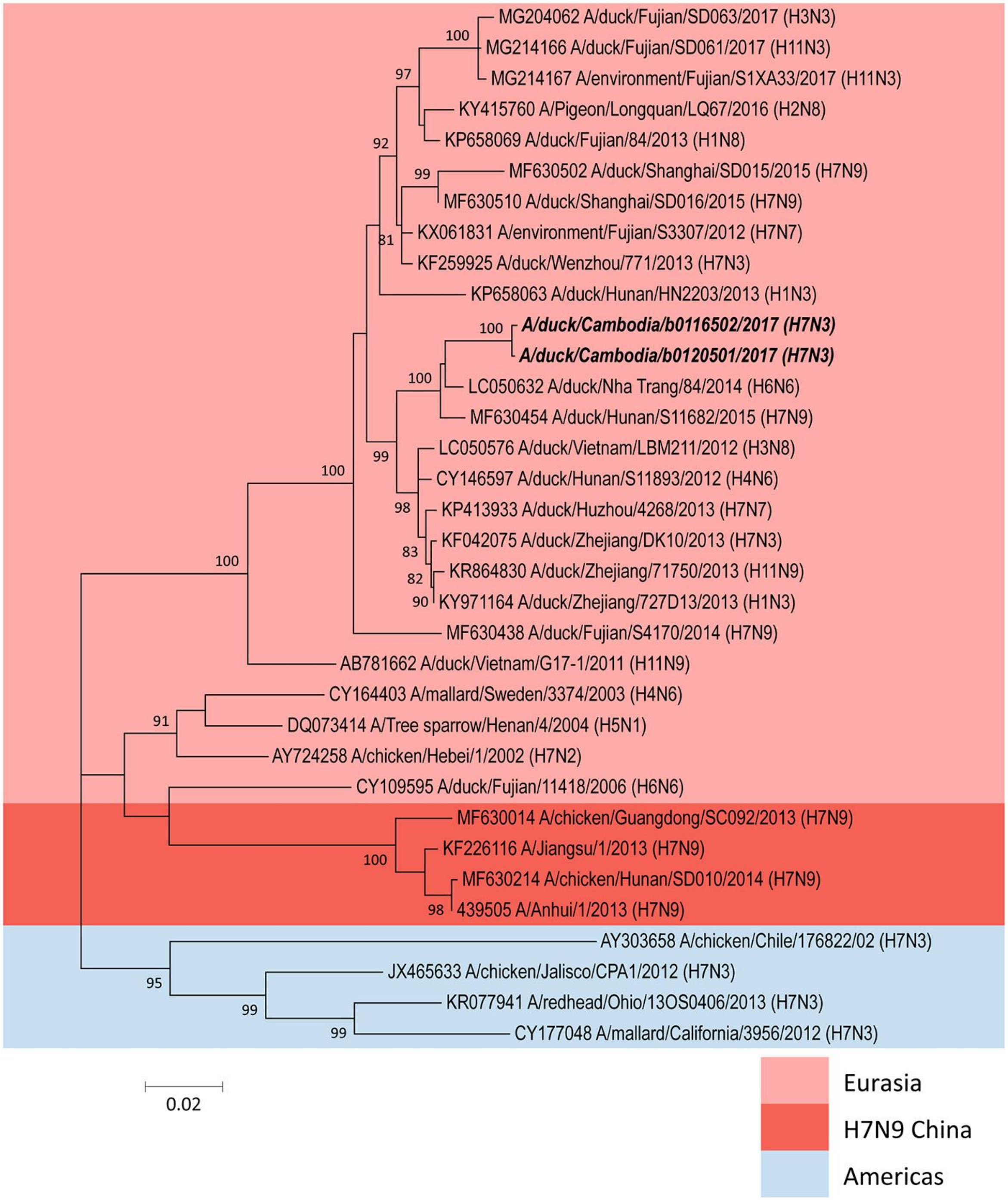

Technical Appendix Figure 6. Phylogenetic analysis (1,497 nt) of the nucleoprotein (NP) gene of viruses isolated from ducks in Kampong Thom province, Cambodia. Trees were generated using the Maximum Likelihood method based on the General Time Reversible model. Scale bars indicate substitutions per site. Bootstrap values (n = 500) >70 are indicated. Cambodian isolates indicated in bold italic. North and South American strains, blue; Eurasian strains, light pink; H7N9 strains from China, red.
